# Diaqua­bis­(4-carb­oxy-2-ethyl-1*H*-imidazole-5-carboxyl­ato-κ^2^ *N* ^3^,*O* ^4^)manganese(II) *N*,*N*-dimethyl­formamide disolvate

**DOI:** 10.1107/S1600536811020071

**Published:** 2011-06-04

**Authors:** Gang Zhang, Yong Wang

**Affiliations:** a Department of Chemistry and Chemical Engineering, Henan University of Urban Construction, Pingdingshan, Henan, People’s Republic of China; b Department of Chemical Engineering, Henan Polytechnic Institute, Nanyang, 473009, People’s Republic of China

## Abstract

In the title compound, [Mn(C_7_H_7_N_2_O_4_)_2_(H_2_O)_2_]·2C_3_H_7_NO, the central Mn^II^ ion, located on an inversion center, is hexa­coordinated by four O atoms from two water mol­ecules and two carboxyl­ate groups, and two N atoms from two 4-carb­oxy-2-ethyl-1*H*-imidazole-5-carboxyl­ate anions in a slightly distorted octa­hedral environment. The complex mol­ecules and solvent mol­ecules are connected *via* N—H⋯O and O—H⋯O hydrogen bonds into a two-dimensional polymeric structure parallel to (001).

## Related literature

For coordination polymers built from 2-ethyl-4,5-imidazole­dicarb­oxy­lic acid, see: Li *et al.* (2011 ▶); Wang *et al.* (2008 ▶); Zhang *et al.* (2010 ▶). For the structure of the analogous Mn^II^ complex with a 5-carb­oxy-2-ethyl-1H-imidazole-4-carboxyl­ate ligand, see: Yan *et al.* (2010 ▶).
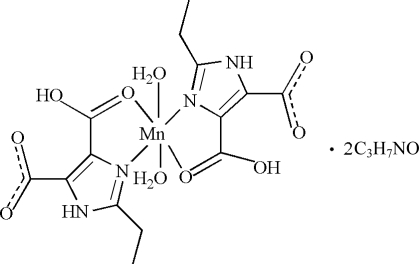


## Experimental

### 

#### Crystal data

[Mn(C_7_H_7_N_2_O_4_)_2_(H_2_O)_2_]·2C_3_H_7_NO
*M* *_r_* = 603.46
Triclinic, 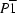

*a* = 7.3246 (2) Å
*b* = 9.0070 (2) Å
*c* = 12.0541 (3) Å
α = 68.841 (1)°
β = 77.780 (1)°
γ = 70.132 (1)°
*V* = 693.89 (3) Å^3^
*Z* = 1
Mo *K*α radiation
μ = 0.54 mm^−1^
*T* = 296 K
0.20 × 0.20 × 0.18 mm

#### Data collection

Bruker APEXII area-detector diffractometer
Absorption correction: multi-scan (*SADABS*; Sheldrick, 1996 ▶) *T*_min_ = 0.899, *T*_max_ = 0.908
5239 measured reflections
2447 independent reflections
2192 reflections with *I* > 2σ(*I*)
*R*_int_ = 0.017

#### Refinement

*R*[*F* ^2^ > 2σ(*F* ^2^)] = 0.034
*wR*(*F* ^2^) = 0.096
*S* = 1.04
2447 reflections
182 parameters
3 restraints
H-atom parameters constrained
Δρ_max_ = 0.33 e Å^−3^
Δρ_min_ = −0.21 e Å^−3^

### 

Data collection: *APEX2* (Bruker, 2007 ▶); cell refinement: *SAINT* (Bruker, 2007 ▶); data reduction: *SAINT*; program(s) used to solve structure: *SHELXS97* (Sheldrick, 2008 ▶); program(s) used to refine structure: *SHELXL97* (Sheldrick, 2008 ▶); molecular graphics: *SHELXTL* (Sheldrick, 2008 ▶); software used to prepare material for publication: *SHELXL97*.

## Supplementary Material

Crystal structure: contains datablock(s) I, global. DOI: 10.1107/S1600536811020071/gk2378sup1.cif

Structure factors: contains datablock(s) I. DOI: 10.1107/S1600536811020071/gk2378Isup2.hkl

Additional supplementary materials:  crystallographic information; 3D view; checkCIF report

## Figures and Tables

**Table 1:** Hydrogen-bond geometry (Å, °)

*D*—H⋯*A*	*D*—H	H⋯*A*	*D*⋯*A*	*D*—H⋯*A*
O1*W*—H2*W*⋯O1^i^	0.80	1.92	2.707 (2)	165
O1*W*—H1*W*⋯O2^ii^	0.82	1.96	2.768 (2)	168
N2—H2⋯O5	0.86	1.89	2.740 (2)	168
O3—H3⋯O2	0.82	1.64	2.462 (2)	179

Symmetry codes: (i) 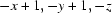; (ii) 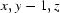.

**Fig. 1.**
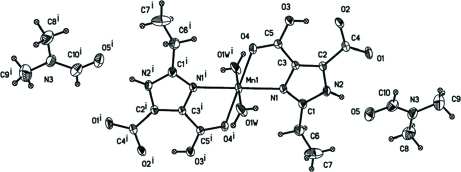
The structure of the title compound showing 30% probability displacement ellipsoids [symmetry codes: (i) 2 - x, -y, -z.]

## supplementary crystallographic information

### Comment

Self-assembly of supramolecular architectures based on imidazole carboxylate ligands has drawn much attention during recent decades. To the best of our knowledge, coordination polymers based on 2-ethyl-4,5-imidazoledicarboxylate ligand has been reperted only in recent years (Wang *et al.*, 2008; Zhang *et al.*, 2010; Li *et al.*, 2011). Herein we report the title compound obtained by the reaction of manganese chloride with 2-ethyl-4,5-imidazoledicarboxylic acid (H_3_EIDC) in a *N*,*N*-dimethylformamide solution under hydrothermal conditions.

The title compound, [Mn(C_7_H_7_N_2_O_4_)_2_(H_2_O)_2_].2C_3_H_7_NO, depicted in Fig. 1. Each Mn^II^ is coordinated by two terminal water molecules, two nitrogen atoms and two oxygen atoms from two chelating 2-ethyl-4,5-imidazoledicarboxylate ligands, generating a distorted octahedral coordination environment. The *N*,*N*-dimethylformamide molecules are connected to the complex molecule *via* hydrogen bond between N2 and O6 atoms (Table 1). In each H_2_EIDC ligand that chelates Mn^II^ ion *via* its N, O atom there is a strong hydrogen bond between the carboxylic and carboxylate groups.

A two-dimensional suramolecular structure is consolidated by intermolecular hydrogen-bonding interactions (N—H···O and O—H···O).

The structure of the title compound is very similar to that formed by 2-propyl-4,5-imidazoledicarboxylate ligand with Mn(II) (Yan *et al.*, 2010).

### Experimental

A mixture of MnCl_2_ (0.5 mmol, 0.06 g) and 2-ethyl-1*H*-imidazole-4,5-dicarboxylic acid (0.5 mmol, 0.95 g) in 15 ml of DMF solution was placed in a 23 ml Teflon-lined reactor, which was heated to 443 K for 4 days, and then cooled to room temperature at a rate of 5 K h^-1^. Crystals of the title compound were obtained by slow evaporation of the solvent at room temperature.

### Refinement

Carboxyl H atoms were located in a difference map but were refined as riding on the parent O atoms with O—H = 0.82 Å and *U*_iso_(H) = 1.5 *U*_eq_(O). Carbon and nitrogen bound H atoms were placed at calculated positions and were treated as riding on the parent C or N atoms with C—H = 0.96 (methyl), 0.97 (methylene) and N—H = 0.86 Å, *U*_iso_(H) = 1.2 or 1.5 *U*_eq_(C, N). H atoms of the water molecule were located in a difference Fourier map and refined as riding with an O—H distance restraint of 0.84 (1) Å, with *U*_iso_(H) = 1.5 *U*_eq_.

### Crystal data

**Table:**

[Mn(C_7_H_7_N_2_O_4_)_2_(H_2_O)_2_]·2C_3_H_7_NO	*Z* = 1
*M**_r_* = 603.46	*F*(000) = 315
Triclinic, *P*1	*D*_x_ = 1.444 Mg m^−^^3^
Hall symbol: -P 1	Mo *K*α radiation, λ = 0.71073 Å
*a* = 7.3246 (2) Å	Cell parameters from 5837 reflections
*b* = 9.0070 (2) Å	θ = 2.8–27.9°
*c* = 12.0541 (3) Å	µ = 0.54 mm^−^^1^
α = 68.841 (1)°	*T* = 296 K
β = 77.780 (1)°	Block, colorless
γ = 70.132 (1)°	0.20 × 0.20 × 0.18 mm
*V* = 693.89 (3) Å^3^	

### Data collection

**Table:**

Bruker APEXII area-detector diffractometer	2447 independent reflections
Radiation source: fine-focus sealed tube	2192 reflections with *I* > 2σ(*I*)
graphite	*R*_int_ = 0.017
φ and ω scans	θ_max_ = 25.0°, θ_min_ = 1.8°
Absorption correction: multi-scan (*SADABS*; Sheldrick, 1996)	*h* = −8→8
*T*_min_ = 0.899, *T*_max_ = 0.908	*k* = −10→10
5239 measured reflections	*l* = −13→14

### Refinement

**Table:**

Refinement on *F*^2^	Primary atom site location: structure-invariant direct methods
Least-squares matrix: full	Secondary atom site location: difference Fourier map
*R*[*F*^2^ > 2σ(*F*^2^)] = 0.034	Hydrogen site location: inferred from neighbouring sites
*wR*(*F*^2^) = 0.096	H-atom parameters constrained
*S* = 1.04	*w* = 1/[σ^2^(*F*_o_^2^) + (0.0528*P*)^2^ + 0.2406*P*] where *P* = (*F*_o_^2^ + 2*F*_c_^2^)/3
2447 reflections	(Δ/σ)_max_ < 0.001
182 parameters	Δρ_max_ = 0.33 e Å^−^^3^
3 restraints	Δρ_min_ = −0.21 e Å^−^^3^

### Special details

**Table:**

Geometry. All e.s.d.'s (except the e.s.d. in the dihedral angle between two l.s. planes) are estimated using the full covariance matrix. The cell e.s.d.'s are taken into account individually in the estimation of e.s.d.'s in distances, angles and torsion angles; correlations between e.s.d.'s in cell parameters are only used when they are defined by crystal symmetry. An approximate (isotropic) treatment of cell e.s.d.'s is used for estimating e.s.d.'s involving l.s. planes.
Refinement. Refinement of *F*^2^ against ALL reflections. The weighted *R*-factor *wR* and goodness of fit *S* are based on *F*^2^, conventional *R*-factors *R* are based on *F*, with *F* set to zero for negative *F*^2^. The threshold expression of *F*^2^ > σ(*F*^2^) is used only for calculating *R*-factors(gt) *etc*. and is not relevant to the choice of reflections for refinement. *R*-factors based on *F*^2^ are statistically about twice as large as those based on *F*, and *R*- factors based on ALL data will be even larger.

### Fractional atomic coordinates and isotropic or equivalent isotropic displacement parameters (Å^2^)

**Table:**

	*x*	*y*	*z*	*U*_iso_*/*U*_eq_	
Mn1	1.0000	0.0000	0.0000	0.03597 (17)	
O1	0.5096 (2)	0.68752 (18)	0.13763 (14)	0.0455 (4)	
O2	0.6923 (2)	0.70548 (17)	−0.03711 (13)	0.0431 (4)	
O3	0.8948 (3)	0.52116 (18)	−0.15377 (14)	0.0483 (4)	
H3	0.8284	0.5834	−0.1154	0.072*	
O4	1.0038 (2)	−0.25098 (18)	0.12795 (13)	0.0420 (4)	
O5	0.3937 (3)	0.3980 (3)	0.39973 (17)	0.0701 (6)	
N1	0.8058 (2)	0.17558 (19)	0.09645 (15)	0.0341 (4)	
N2	0.6175 (3)	0.3399 (2)	0.19823 (15)	0.0362 (4)	
H2	0.5413	0.3718	0.2554	0.043*	
N3	0.2062 (3)	0.5787 (3)	0.49596 (18)	0.0519 (5)	
C1	0.6958 (3)	0.1811 (3)	0.19781 (18)	0.0387 (5)	
C2	0.6797 (3)	0.4415 (2)	0.09255 (17)	0.0298 (4)	
C3	0.7965 (3)	0.3374 (2)	0.02988 (17)	0.0292 (4)	
C4	0.6210 (3)	0.6249 (2)	0.06428 (18)	0.0337 (4)	
C5	0.9031 (3)	0.3697 (2)	−0.09070 (17)	0.0330 (4)	
C6	0.6624 (4)	0.0344 (3)	0.2986 (2)	0.0594 (7)	
H6A	0.7410	−0.0656	0.2792	0.071*	
H6B	0.7080	0.0322	0.3693	0.071*	
C7	0.4567 (6)	0.0304 (5)	0.3278 (4)	0.1004 (13)	
H7A	0.3757	0.1320	0.3423	0.151*	
H7B	0.4466	−0.0620	0.3980	0.151*	
H7C	0.4145	0.0187	0.2620	0.151*	
C8	0.2127 (5)	0.4520 (5)	0.6120 (2)	0.0766 (9)	
H8A	0.2852	0.3454	0.6029	0.115*	
H8B	0.0822	0.4508	0.6464	0.115*	
H8C	0.2748	0.4761	0.6634	0.115*	
C9	0.1012 (6)	0.7462 (5)	0.4934 (4)	0.1007 (13)	
H9A	0.0953	0.8174	0.4120	0.151*	
H9B	0.1668	0.7829	0.5350	0.151*	
H9C	−0.0288	0.7505	0.5315	0.151*	
C10	0.2976 (4)	0.5403 (4)	0.3999 (2)	0.0603 (7)	
H10	0.2897	0.6254	0.3272	0.072*	
O1W	0.7541 (2)	0.0082 (2)	−0.07777 (18)	0.0579 (5)	
H1W	0.7407	−0.0852	−0.0560	0.087*	
H2W	0.6611	0.0895	−0.0898	0.087*	

### Atomic displacement parameters (Å^2^)

**Table:**

	*U*^11^	*U*^22^	*U*^33^	*U*^12^	*U*^13^	*U*^23^
Mn1	0.0376 (3)	0.0226 (2)	0.0446 (3)	−0.00243 (18)	0.00243 (19)	−0.01653 (19)
O1	0.0533 (9)	0.0297 (8)	0.0465 (9)	0.0017 (7)	0.0006 (7)	−0.0197 (7)
O2	0.0565 (9)	0.0226 (7)	0.0465 (9)	−0.0090 (7)	0.0017 (7)	−0.0127 (7)
O3	0.0653 (11)	0.0272 (8)	0.0415 (8)	−0.0125 (7)	0.0157 (7)	−0.0109 (7)
O4	0.0489 (9)	0.0306 (8)	0.0404 (8)	−0.0073 (7)	0.0118 (7)	−0.0173 (6)
O5	0.0795 (13)	0.0729 (14)	0.0570 (11)	−0.0180 (11)	0.0185 (10)	−0.0372 (10)
N1	0.0374 (9)	0.0228 (8)	0.0362 (9)	−0.0041 (7)	0.0038 (7)	−0.0110 (7)
N2	0.0416 (9)	0.0293 (9)	0.0313 (9)	−0.0032 (7)	0.0044 (7)	−0.0131 (7)
N3	0.0464 (11)	0.0616 (13)	0.0494 (12)	−0.0141 (10)	0.0077 (9)	−0.0278 (10)
C1	0.0434 (12)	0.0274 (11)	0.0364 (11)	−0.0047 (9)	0.0022 (9)	−0.0086 (9)
C2	0.0311 (10)	0.0254 (10)	0.0317 (10)	−0.0042 (8)	−0.0032 (8)	−0.0112 (8)
C3	0.0304 (9)	0.0224 (9)	0.0331 (10)	−0.0047 (7)	−0.0007 (8)	−0.0108 (8)
C4	0.0360 (10)	0.0267 (10)	0.0382 (11)	−0.0031 (8)	−0.0064 (9)	−0.0141 (9)
C5	0.0358 (10)	0.0265 (10)	0.0346 (10)	−0.0080 (8)	0.0020 (8)	−0.0114 (8)
C6	0.0736 (18)	0.0351 (13)	0.0485 (14)	−0.0099 (12)	0.0116 (12)	−0.0043 (11)
C7	0.109 (3)	0.093 (3)	0.088 (2)	−0.064 (2)	−0.007 (2)	0.014 (2)
C8	0.091 (2)	0.096 (2)	0.0454 (15)	−0.0368 (19)	0.0138 (15)	−0.0275 (16)
C9	0.085 (2)	0.084 (3)	0.129 (3)	−0.006 (2)	0.011 (2)	−0.058 (3)
C10	0.0607 (16)	0.072 (2)	0.0449 (14)	−0.0210 (14)	0.0073 (12)	−0.0204 (13)
O1W	0.0502 (9)	0.0266 (8)	0.0991 (14)	−0.0010 (7)	−0.0228 (9)	−0.0230 (9)

### Geometric parameters (Å, °)

**Table:**

Mn1—O1W^i^	2.1683 (17)	C1—C6	1.489 (3)
Mn1—O1W	2.1683 (17)	C2—C3	1.369 (3)
Mn1—O4^i^	2.2244 (15)	C2—C4	1.484 (3)
Mn1—O4	2.2244 (15)	C3—C5	1.475 (3)
Mn1—N1^i^	2.2302 (15)	C5—O4^i^	1.238 (2)
Mn1—N1	2.2302 (15)	C6—C7	1.483 (5)
O1—C4	1.228 (2)	C6—H6A	0.9700
O2—C4	1.281 (3)	C6—H6B	0.9700
O3—C5	1.287 (2)	C7—H7A	0.9600
O3—H3	0.8200	C7—H7B	0.9600
O4—C5^i^	1.238 (2)	C7—H7C	0.9600
O5—C10	1.235 (4)	C8—H8A	0.9600
N1—C1	1.321 (3)	C8—H8B	0.9600
N1—C3	1.373 (2)	C8—H8C	0.9600
N2—C1	1.350 (3)	C9—H9A	0.9600
N2—C2	1.365 (3)	C9—H9B	0.9600
N2—H2	0.8600	C9—H9C	0.9600
N3—C10	1.309 (3)	C10—H10	0.9300
N3—C9	1.434 (4)	O1W—H1W	0.8200
N3—C8	1.449 (4)	O1W—H2W	0.8047
			
O1W^i^—Mn1—O1W	180.00 (10)	O1—C4—C2	118.76 (18)
O1W^i^—Mn1—O4^i^	90.79 (6)	O2—C4—C2	116.00 (17)
O1W—Mn1—O4^i^	89.21 (6)	O4^i^—C5—O3	122.34 (18)
O1W^i^—Mn1—O4	89.21 (6)	O4^i^—C5—C3	119.06 (18)
O1W—Mn1—O4	90.79 (6)	O3—C5—C3	118.59 (17)
O4^i^—Mn1—O4	180.00 (11)	C7—C6—C1	115.0 (2)
O1W^i^—Mn1—N1^i^	90.95 (6)	C7—C6—H6A	108.5
O1W—Mn1—N1^i^	89.05 (6)	C1—C6—H6A	108.5
O4^i^—Mn1—N1^i^	104.47 (5)	C7—C6—H6B	108.5
O4—Mn1—N1^i^	75.53 (5)	C1—C6—H6B	108.5
O1W^i^—Mn1—N1	89.05 (6)	H6A—C6—H6B	107.5
O1W—Mn1—N1	90.95 (6)	C6—C7—H7A	109.5
O4^i^—Mn1—N1	75.53 (5)	C6—C7—H7B	109.5
O4—Mn1—N1	104.47 (5)	H7A—C7—H7B	109.5
N1^i^—Mn1—N1	180.00 (7)	C6—C7—H7C	109.5
C5—O3—H3	109.5	H7A—C7—H7C	109.5
C5^i^—O4—Mn1	115.88 (12)	H7B—C7—H7C	109.5
C1—N1—C3	106.19 (16)	N3—C8—H8A	109.5
C1—N1—Mn1	142.61 (14)	N3—C8—H8B	109.5
C3—N1—Mn1	111.18 (12)	H8A—C8—H8B	109.5
C1—N2—C2	108.45 (17)	N3—C8—H8C	109.5
C1—N2—H2	125.8	H8A—C8—H8C	109.5
C2—N2—H2	125.8	H8B—C8—H8C	109.5
C10—N3—C9	122.6 (3)	N3—C9—H9A	109.5
C10—N3—C8	120.8 (3)	N3—C9—H9B	109.5
C9—N3—C8	116.6 (3)	H9A—C9—H9B	109.5
N1—C1—N2	110.34 (18)	N3—C9—H9C	109.5
N1—C1—C6	125.5 (2)	H9A—C9—H9C	109.5
N2—C1—C6	124.14 (19)	H9B—C9—H9C	109.5
N2—C2—C3	105.31 (17)	O5—C10—N3	124.1 (3)
N2—C2—C4	122.29 (17)	O5—C10—H10	118.0
C3—C2—C4	132.40 (18)	N3—C10—H10	118.0
C2—C3—N1	109.70 (16)	Mn1—O1W—H1W	109.5
C2—C3—C5	132.07 (18)	Mn1—O1W—H2W	120.7
N1—C3—C5	118.21 (16)	H1W—O1W—H2W	120.8
O1—C4—O2	125.24 (19)		

Symmetry codes: (i) −*x*+2, −*y*, −*z*.

### Hydrogen-bond geometry (Å, °)

**Table:**

*D*—H···*A*	*D*—H	H···*A*	*D*···*A*	*D*—H···*A*
O1W—H2W···O1^ii^	0.80	1.92	2.707 (2)	165
O1W—H1W···O2^iii^	0.82	1.96	2.768 (2)	168
N2—H2···O5	0.86	1.89	2.740 (2)	168
O3—H3···O2	0.82	1.64	2.462 (2)	179

Symmetry codes: (ii) −*x*+1, −*y*+1, −*z*; (iii) *x*, *y*−1, *z*.
